# Angiogenesis and Immunity in Renal Carcinoma: Can We Turn an Unhappy Relationship into a Happy Marriage?

**DOI:** 10.3390/jcm9040930

**Published:** 2020-03-28

**Authors:** Alessia Mennitto, Veronica Huber, Raffaele Ratta, Pierangela Sepe, Filippo de Braud, Giuseppe Procopio, Valentina Guadalupi, Mélanie Claps, Marco Stellato, Elena Daveri, Licia Rivoltini, Elena Verzoni

**Affiliations:** 1 Department of Medical Oncology, Fondazione IRCCS Istituto Nazionale dei Tumori, 20133 Milan, Italy; pieranela.sepe@istitutotumori.mi.it (P.S.); filippo.debraud@istitutotumori.mi.it (F.d.B.); giuseppe.procopio@istitutotumori.mi.it (G.P.); valentina.guadalupi@istitutotumori.mi.it (V.G.); melanie.claps@istitutotumori.mi.it (M.C.); marco.stellato@istitutotumori.mi.it (M.S.); elena.verzoni@istitutotumori.mi.it (E.V.); 2 Unit of Immunotherapy of Human Tumors, Fondazione IRCCS Istituto Nazionale dei Tumori, 20133 Milan, Italy; veronica.huber@istitutotumori.mi.it (V.H.); elena.daveri@istitutotumori.mi.it (E.D.); licia.rivoltini@istitutotumori.mi.it (L.R.); 3 Oncology and Supportive Care Department, Hôpital Foch, 40 Rue Worth, 92151 Suresnes, France; r.ratta@hopital-foch.com; 4 Department of Oncology and Hemato-Oncology, University of Milan, 20122 Milan, Italy

**Keywords:** metastatic renal cell carcinoma, angiogenesis, immunotherapy, tyrosine-kinase inhibitors, immunomodulation, immune checkpoint inhibitors

## Abstract

The frontline treatment options for patients with metastatic renal cell carcinoma (mRCC) are evolving rapidly since the approval of combination immunotherapies by the U.S. Food and Drug Administration (USFDA) and the European Medicines Agency (EMA). In particular, in combination with vascular endothelial growth factor receptor (VEGFR) tyrosine-kinase inhibitors (TKIs), immune checkpoint inhibitors (ICIs) have significantly improved the outcome of patients with mRCC compared to TKI monotherapy. Here, we review the preclinical data supporting the combination of ICIs with VEGFR TKIs. The VEGF-signaling inhibition could ideally sustain immunotherapy through a positive modulation of the tumor microenvironment (TME). Antiangiogenetics, in fact, with their inhibitory activity on myelopoiesis that indirectly reduces myeloid-derived suppressor cells (MDSCs) and regulatory T cells’ (Tregs) frequency and function, could have a role in determining an effective anti-tumor immune response. These findings are relevant for the challenges posed to clinicians concerning the clinical impact on treatment strategies for mRCC.

## 1. Introduction

Renal cell carcinoma (RCC) represents the seventh most common cancer, with 330,000 cases diagnosed and more than 140,000 deaths per year worldwide [1].

Over the last decades, the therapeutic scenario of metastatic RCC (mRCC) has radically changed. Until 2005, interferon alfa (IFN-α) and high-dose interleukin-2 (HD IL-2) were the standard of care for the treatment of mRCC [2,3]. However, their impact on immune-escape mechanisms was limited, and responses to treatments were often poor, not durable and associated with a bad tolerability [4].

Recently, a better understanding of the biological and molecular basis of RCC has led to the development and approval of new targeted agents: the majority of these drugs are directed against the vascular endothelial growth factor (VEGF)/VEGF receptors (VEGFRs) pathway (bevacizumab, sorafenib, sunitinib, pazopanib, axitinib and cabozantinib) [5,6,7,8,9]; the mammalian target of the rapamycin (mTOR) pathway (everolimus and temsirolimus) [10,11] and the PD-1/PD-L1 pathway (nivolumab) [12,13].

By targeting endothelial cell proliferation, tumor angiogenesis and growth and by stimulating the immune system, these drugs have improved clinical outcomes. Indeed, response rates (RR) exceed 30%, and median overall survival (mOS) is almost two years, depending on patient risk profile, the type of treatment and other clinical variables [14].

Moreover, clinical trials have shown that the combination of VEGFR tyrosine-kinase inhibitors (TKIs) and antibodies targeting PD-1 and PD-L1 present stronger activity when compared to TKI monotherapy [15].

RCC represents a paradigmatic example of a tumor with diverse host reactions taking place, allowing to study how these responses might influence tumor growth. Indeed, RCC is featured by profound neoangiogenic processes, mostly driven by oncogenic hallmarks linked to the von-Hippel Lindau (VHL) gene. On the other hand, RCC is also a quite immunogenic cancer, displaying an extraordinarily rich and heterogeneous immune infiltrate, as depicted in the excellent papers recently published on RCC immune atlas [16]. Understanding how angiogenesis and immunity do crosstalk within the tumor microenvironment (TME) and influence each other is a key point to guide therapeutic choices and sequences in a patient-tailored approach to maximize clinical efficacy.

## 2. Mutually Exclusive Features of Clear Cell Renal Carcinoma Microenvironment

Together with melanoma, RCC has been considered for decades the most immunogenic among human cancer types. Its rich microenvironment, characterized by a plethora of immune cells encompassing T cells, myeloid cells, macrophages, granulocytes, natural killer (NK) cells and other subsets [17], has for long been considered a unique feature. In particular, kidney cancer has been reported to display the highest level of T cell infiltration score among 19 different tumor types [18], indicating the active reaction of the host immune defenses to restrain tumor growth. T cells usually are triggered by the expression on tumor cells of immunogenic determinants (represented by short fragments of antigenic proteins bound to HLA-class I or II molecules) stemming from the altered protein repertoire. While these alterations often originate from DNA mutations related to cancer genetic instability, in the case of RCC, the number of somatic missense mutations is quite low, followed only by thyroid cancer and lower grade glioma [18]. Instead, tumor-specific neoantigens are generated by the abundance of insertions-and-deletions detected in RCC cell DNA [19] and by the highly functional antigen-processing-machinery genes that favor antigen presentation by cancer cells [20].

**Figure 1:**
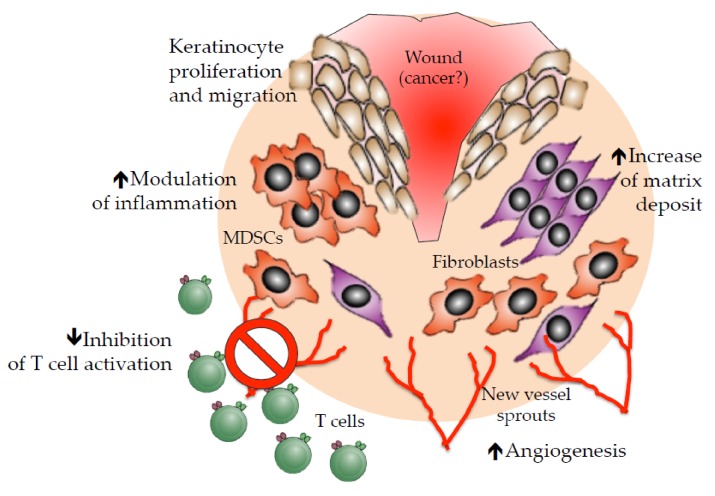
The link between angiogenesis and immunosuppression in the physiological process of wound healing has some homologies with cancer. MDSCs = myeloid-derived suppressor cells.

This remarkably immunogenic scenario is also mirrored at the transcriptional level. Indeed, mRNA signatures identified three main clusters on the basis of the immune infiltrate: tumors featured by T cell-enriched vs. non-infiltrated signatures and a third cluster with an intermediate and heterogeneous milieu. T cell-enriched tumors show enhanced expression of genes involved in full-fledged adaptive immunity (IFNs, granzyme, perforin and Th1 cytokines), including the immune checkpoints CTLA4, PD-1, PDL-1, TIM3 and LAG3, as a sign of engaged immune escape. In contrast, pathways involved in angiogenic processes are specifically detected in non-immune-infiltrated lesions [18,21]. These latter are also enriched in transcriptomic signatures associated with immunosuppressive pathways involving myeloid cells and innate immunity, mesenchymal transition and fibrosis [21]. These apparently mutually exclusive pictures, where T cell immune infiltrate does not coexist with neoangiogenic processes, stem from the well-known evidence that angiogenesis mediates potent suppression on immunity, both directly, through the inhibitory activity of VEGF, and indirectly, via the induction of myeloid and lymphoid immunoregulatory cells. Interestingly, the link between angiogenesis and immunosuppression has its origin in the physiological process of wound healing (Figure 1). Here, angiogenesis and stroma remodeling promote the accrual of myeloid-derived suppressor cells (MDSCs) and regulatory T cells (Tregs) to avoid a potential onset of autoreactivation by T and B cells, which might hamper tissue repair [22,23].

The possible implications of these interconnected processes on the therapeutic strategies to control RCC are clear and explain why this tumor responds to treatments rescuing antitumor immunity only when expressing T cell-enriched signatures, while antiangiogenic approaches are effective in T cell-depleted cancers [24].

## 3. Crosstalk between Angiogenesis and Immunity

Angiogenesis plays a pivotal role in the development and progression of RCC. Indeed, about 60% of RCC tumors present an inactivated VHL tumor suppressor gene through somatic mutations (∼50% of cases) or promoter methylations (∼10%) [25]. This results in a constitutive expression of the transcription factor hypoxia-factor (HIF)1α and in the overexpression of proangiogenic VEGF, platelet-derived growth factor (PDGF), erythropoietin (EPO) and insulin-like growth factor 2 (IGF2) [26]. This imbalance of pro- and antiangiogenic factors leads to multiple structural and functional abnormalities in blood vessels. In fact, these are irregularly shaped, tortuous and hyperpermeable, often covered by anergic endothelial cells, and without pericytes [27,28]. All these changes result in an abnormal blood flow with consequent tumor cell extravasion, intratumoral T-cell infiltration and altered antineoplastic drug delivery [29]. These mechanisms involved in tumorigenesis of RCC have been confirmed by RNA and protein microarray studies showing that the upregulation of VEGF correlated with high microvessel density and advanced stage tumor progression and poor prognosis [30,31].

In addition to their angiogenic properties, the constitutive activation of HIF1α and the increased levels of VEGF induce the release of immunosuppressive factors, such as transforming growth factor β (TGF-β), PD-L1 and VEGF itself. These inhibit the maturation and recruitment of dendritic cells (DCs) through the nuclear factor κB (NF-κB)-dependent pathway [32,33], activate inflammatory cells with immunosuppressive functions, such as MDSCs [34], and increase the infiltration of tumor-associated macrophages (TAMs) [35]. DCs are antigen-presenting cells that, under physiological conditions, promote tolerance to self-antigens through the control of Tregs, specialized T cells with immunosuppressive functions [36]. However, in cancer patients, Tregs mediate tumor immune evasion through the expression of inhibitory molecules on their surface and the release of cytokines that suppress effector T cells, NK cells and other leukocytes involved in anticancer immunity [37,38]. Immune dysfunction is well-described in RCC patients who experience a shift from a type-1 mediated CD4^+^ T-cell response producing IFN-γ (involved in the effective antitumor immune response), to a type-2 cytokine response implicated in the humoral immunity [39,40]. The accumulation of Tregs in the TME correlates with an unfavorable prognosis in several neoplasms [41,42]. MDSCs are progenitors of granulocytes and monocytes, physiologically present in the bone marrow and peripheral blood. In RCC and generally in cancer patients, tumor cells secrete multiple cytokines (e.g., CXCL-8 and CCL-2/3/4/5), which recruit MDSCs to TME and inhibit their maturation [43]. These mechanisms enhance neoangiogenesis via the VEGF pathway, immunosuppression and mesenchymal transition [44,45]. MDSCs suppress antitumor immunity through different mechanisms: they inhibit the activation of CD4^+^ and CD8^+^ lymphocytes causing the arrest of their cell cycle in the G0/G1 phase [46], reduce the proliferation and infiltration of effector T cells [47], further stimulate Tregs function [48] and drive monocytes’ differentiation toward activated M2 macrophages [49]. Moreover, VEGF-driven angiogenesis downregulates vascular adhesion molecules on tumor-infiltrating endothelial cells in vivo and in vitro, reducing the infiltration of cytolytic effector leukocytes into tumors [50].

On the other hand, the immune microenvironment supports angiogenesis, too. Immune cells cooperate and synergize with stromal and cancer cells in stimulating the growth, migration and activation of endothelial cells through the production and release of a large spectrum of proangiogenic mediators, leading to blood vessel formation [51]. DCs express both VEGFR-1 and VEGFR-2 and can release pro- or antiangiogenic mediators when exposed to different combinations of cytokines. In the presence of prostaglandin E2 (PG-E2) or IL-10, DCs show an angiogenesis-promoting phenotype through the secretion of VEGF [51,52]. Eosinophils, neutrophils and TAMs have the capacity to generate new vessels through the release of a rich armamentarium of growth factors, including GM-CSF; nerve growth factor (NGF); angiogenin; TNF-α; IL-8; VEGF; fibroblast growth factor 2 (FGF-2) [53,54,55,56,57]; chemokines like CXCL-3, -4, -8, -9, -10 and CCL2-5 and expression of the cognate receptors (CXCR-2, -4 and -12) [58,59]. Moreover, these cells express angiogenesis-modulating enzymes such as cycloxygenase-2 (COX-2) and metalloproteinases (MMP-2, -7, -9 and -12) implicated in the release and mobilization of VEGF. This can destabilize the vasculature acting on matrix degradation, causing tumor migration and metastases [60]. Lastly, T cells are also indirectly involved in neoangiogenesis. Indeed, they bind and vehicle VEGF through the body after the acquisition of neuropilin-1 (NRP1) by trogocytosis from DCs. This transmembrane protein is expressed on neuronal and endothelial cells where it plays a crucial role in guiding axons and regulating angiogenesis [61]. Large amounts of preclinical data undoubtedly demonstrated that VEGF controls immune tolerance and surveillance in cancer. In mice genetically knocked-out for VEGF expression, a broad activation of immune-related molecules, such as chemokines mediating T-cell accrual, signaling pathways involved in T-cell activation and factors responsible for T-cell cytotoxic activity, can be detected in tumor lesions [62]. Most importantly, large parts of the therapeutic activity of antiangiogenics in murine settings rely on the rescue of antitumor immunosurveillance, as indicated by the evidence that the VEGF blockade loses its therapeutic efficacy in CD3 T-cell-depleted animals [62]. Furthermore, VEGF-A induces the upregulation of multiple immune checkpoints, such as PD-1, CTLA4, TIM3 and LAG3, in tumor-infiltrating CD8^+^ T cells, and VEGF-silencing potentiates tumor control mediated by the PD-1 blockade [63]. Quite recent studies demonstrate that also emerging immunotherapeutic molecules like anti-CD40 antibodies benefit from the concomitant blocking of angiogenesis, albeit a dual Ang2 and VEGF-A antagonism is required to favor intratumoral redistribution of CD8^+^ T cells [64].

Altogether, these data provide further support to the evidence that angiogenesis and immunity are processes strictly interconnected and mutually exclusive in the absence of therapeutic intervention and that interruption of angiogenesis may favor the rescue of host immune defenses mediated by immunotherapy.

## 4. Effects of TKIs on Immunity

One of the targets of TKIs is VEGFR, which, when blocked, fails to activate endothelial cells to generate new vessels in the tumor, normalizing angiogenesis and restoring an adequate tissue perfusion. The consequence is a normalization of the TME, a reduced release of growth factors and the recovery of host immunity [65]. Increasing evidence shows that antiangiogenic molecules overcome various immunosuppressive networks.

Sunitinib has been shown to reduce the number of systemic and intratumoral Tregs starting from the first course of therapy and, with each cycle thereafter, resulting in an improved type-1 cytokine response. In particular, Adotevi and colleagues have observed that patients with higher levels of Tregs at baseline are more likely to present a reduction after the second or third cycle of therapy than patients presenting lower pretreatment levels of Tregs [66]. An in vitro study showed that sunitinib did not suppress the expansion of Tregs over a 14-day incubation period, suggesting that sunitinib has an indirect effect on the Treg population in vivo [67]. A possible explanation is that the activity of Tregs, which express VEGFR2 on their surface, is mediated by VEGF [68] or by MDSCs, whose percentage is reduced during sunitinib-based treatment with a synchronous higher production of IFN-γ by T lymphocytes [69]. The depletion of MDSCs induced by sunitinib seems to be mediated by the inhibition of VEGF, c-Kit and Stat3, which, when activated, promote various immunosuppressive mechanisms (e.g., blockade of DC maturation and release of IL-10) and the expression of angiogenic and metastatic factors, inducing cancer cell resistance to the apoptotic activity of cytotoxic T cells [70,71].

The immune-modulating activities of sorafenib are less clear. In contrast to sunitinib, some detrimental effects have been described. Indeed, this drug seems to interfere with the maturation and the antigen presentation properties of DCs through the downregulation of a major histocompatibility complex and costimulatory molecules and the decreased production of immunostimulatory cytokines [72]. On the other hand, sorafenib reduces the percentage of circulating and tumor-infiltrating Tregs [73,74] and inhibits the activation of macrophages [75], shifting the immune balance to a more stimulatory setting. Similar to sunitinib, pazopanib also neutralizes the immunosuppressive effects of VEGF by reducing the expression of VEGFR1 and VEGFR2, which affect final DC maturation [76,77]. An in vitro study reported that DCs from pazopanib-treated patients expressed more activation markers HLA-DR and CCR7 and less PD-L1 as compared to DCs generated from sunitinib-treated patients [78]. Moreover, pazopanib decreases the amount of MDSCs, CD14^+^ monocytes and Tregs and triggers T-cell memory Th1 response, CD8^+^ lymphocytes and NK effectors [79]. Sunitinib and pazopanib have a similar pattern of receptor recognition but different affinities for VEGFR1. Consequently, a diversity of immune modulation effects of the two drugs has been observed. In particular, pazopanib displayed more activity in restoring the activation of DCs, whereas sunitinib is more efficient in potentiating the antitumor effector cells by eliminating immunosuppression in the TME [80]. Lastly, the immunomodulatory functions of cabozantinib occur through the reduction of MDSCs and the increased activity of circulating cytotoxic NK and CD8^+^ T cells, acting on both direct and antibody-mediated tumor killing [81].

In light of the immunomodulatory potential of antiangiogenics, combining anti-VEGF/VEGFR agents with ICIs has emerged as a strategy to synergize and potentiate the therapeutic efficacy of these treatments. However, it should be mentioned that, despite no side-by-side study testing the immunomodulating properties of the diverse antiangiogenics in a clinical setting having been performed, preclinical studies suggest that subtle but clinically relevant differences might exist [82,83,84]. One representative example is the key study of Stehle et al. [85] showing, albeit only in vitro, that some TKI-based antiangiogenics—for instance, sorafenib and sunitinib—exert marked suppressive activity on T-cell proliferation and function, while others (e.g., axitinib) display a more neutral effect. This clearly indicates that, in combinatory approaches with immunotherapy, a more careful mechanism-driven choice of the drugs to pair in clinical trials would be indicated.

## 5. Conclusions

A major part of relevant information about human TME of the last decade undoubtedly derive from the study of RCC. Nevertheless, the application of these key principles that would help designing drug sequences and combinations is not so readily translated into the clinical setting. Quite recent trials, such as, for instance, the IMmotion151 [24], have indeed exploited tumor transcriptional signatures to predict responses to antiangiogenics with or without ICIs, retrospectively, but the approach is still in its infancy.

Based on the biological scenarios depicted in this review, some potential considerations could be made. Antiangiogenics with pleiotropic immunomodulating properties (like pazopanib, cabozantinib and axitinib), in combination with immunotherapeutics, could be preferred to molecules showing more immunosuppressive effects on T-cell function (e.g., sunitinib and sorafenib). Considering instead drug sequences, the administration of ICIs at the failure of antiangiogenics could be penalized by the accrual of immunosuppressive cells such as MDSCs, which associate with resistance to the VEGF blockade and an immune hostile milieu always paralleling progressing cancers [86,87].

The efficacy of antiangiogenics at progression to ICIs might instead depend on the resistance mechanisms that might vary at individual patient levels [88,89]. If resistance is linked to the tumor loss of molecular determinants required for T-cell recognition, drugs such as pazopanib and cabozantinib that induce the activation of NK cells [79,81], so abundantly represented in RCC TME [38], could be effective.

The immunomodulating property of antiangiogenic TKIs, mostly due to their direct inhibitory activity on myelopoiesis indirectly reducing MDSC frequency and function [79,81], could also be exploited in a more innovative fashion based on their activity kinetics. Indeed, systemic immunomodulation occurs rapidly but transiently, being detectable in peripheral blood at three months but mostly disappearing at six months [79]. This effect could be exploited to conceive the use of antiangiogenics as a sort of “conditioning” treatment to favor a response to immunotherapy. Indeed, the administration of immune checkpoint blockers at TKI-induced MDSC nadir, coinciding also with an increase of PD-1^+^-activated T and NK cells, holds promises to potentiate clinical efficacy significantly. Sequential alternate therapeutic schedules are not so common in the treatment of solid tumors, but they are often applied for patient conditioning in hemato-oncology [90,91,92]. The use of alternate schedules based on a defined biological rationale could help maximize efficacy while reducing the risk of resistance and possibly also of toxicity.

Thanks to the availability of therapeutic drugs with defined immunomodulating properties, RCC could represent an ideal clinical setting to investigate whether tailoring treatments based on the immune profile of individual patients and tumors might contribute to increased disease control in the context of a more innovative view of precision oncology.
